# Influence of Urbanization on Epiphytic Bacterial Communities of the *Platanus × hispanica* Tree Leaves in a Biennial Study

**DOI:** 10.3389/fmicb.2019.00675

**Published:** 2019-04-04

**Authors:** Jordan Espenshade, Sofie Thijs, Stanislaw Gawronski, Hannelore Bové, Nele Weyens, Jaco Vangronsveld

**Affiliations:** ^1^Centre for Environmental Sciences, Hasselt University, Diepenbeek, Belgium; ^2^Faculty of Horticulture, Biotechnology and Landscape Architecture, Warsaw University of Life Sciences, Warsaw, Poland; ^3^Biomedical Research Institute, Hasselt University, Diepenbeek, Belgium; ^4^Centre for Surface Chemistry and Catalysis, KU Leuven, Leuven, Belgium; ^5^Department of Plant Physiology, Faculty of Biology and Biotechnology, Maria Curie-Skłodowska University, Lublin, Poland

**Keywords:** urban microbiome, phyllosphere-inhabiting bacteria, phylloremediation, biodiversity, plant-microbe interactions, particulate matter, black carbon

## Abstract

The aerial surfaces of plants harbor diverse communities of microorganisms. The rising awareness concerning the potential roles of these phyllosphere microbiota for airborne pollutant remediation and plant growth promotion, advocates for a better understanding of their community structure and dynamics in urban ecosystems. Here, we characterized the epiphytic microbial communities on leaves of *Platanus × hispanica* trees in the city centre of Hasselt (Belgium), and the nearby forest area of Bokrijk, Genk (Belgium). We compared the influences of season, site, and air pollutants concentration variations on the tree’s phyllosphere microbiome by determining the intra- and inter-individual variation in leaf bacterial communities. High-throughput amplicon sequencing of the 16S rRNA gene revealed large variation in the bacterial community structure and diversity throughout the years but also allowed to discriminate an environment effect on community assembly. Partial drivers for this environment effect on composition can be correlated with the huge differences in ultrafine particulate matter (UFP) and black carbon on the leaves. A change in bacterial community composition was noted for trees growing in the city center compared to the natural site, and also more human-associated genera were found colonizing the leaves from the city center. These integrated results offer an original and first insight in the *Platanus* phyllomicrobiota, which can offer new opportunities to use phyllosphere microorganisms to enhance air pollution degradation.

## Introduction

Microorganisms associated with the aerial structures of plants, otherwise known as the phyllosphere, are receiving greater attention in urban microbiome research (King, 2014). An important part of future urban planning is minimizing environmental degradation for the health and safety of the residents. The “biodiversity hypothesis” connects evidence between the rise of non-communicable diseases in urbanized populations to the decrease of environmental biodiversity (Haahtela et al., 2013; Rook, 2013). In 2018, 55% of the world’s population live in urban environments and this is expected to increase in the future with rising population growth (United Nations, 2018). In China, already 60 % of its population lives in cities, and more than 100 Chinese cities count above 1 million people (The Guardian, 2017). Urban residents are more likely to be exposed to higher levels of air pollution, amongst which traffic related sources of pollution contribute an important part, and which leads to lung and cardiovascular diseases and premature death. In the past few years, several European cities have tried to regulate the volume or type of vehicles allowed within the city limits, but annual concentrations of air pollution continue to exceed the WHO guidelines (Holman et al., 2015). Besides regulation, plans to mitigate exposure to air pollution and to promote urban green coverage for scavenging airborne pollutants (fine dust, soot particles, PAHs) each represent important efforts to combat this ubiquitous problem.

To enhance the capacity of vegetation to intercept airborne pollutants, the selection of both the appropriate plant species and their associated phyllospheric microbiome are important, as both influence the performance of phylloremediation (Weyens et al., 2015). Depending on the species, many plants have the physical characteristics to capture significant amounts of particulate matter (PM), thereby improving local air quality (Sæbø et al., 2012; Nowak et al., 2013; Popek et al., 2013). Phyllospheric microorganisms can enhance this pollutant sequestration effect by improving plant growth and health, via various direct and indirect mechanisms such as stress reduction by interfering with plant ethylene levels, improving drought resistance, improving protection from herbivore stress, salinity, and promoting plant growth through the synthesis of plant-growth hormones, and improving nutrient uptake (Vorholt, 2012; Weyens et al., 2015; Saleem et al., 2017). In addition, epiphytic microorganisms can enhance xenobiotic pollutant transformations *ex planta* and contribute to nutrient uptake and conversion, while the endophytic community is important for transforming organic compounds *in planta*, thus reducing their toxicity (Barac et al., 2004; Taghavi et al., 2005; Weyens et al., 2009a,b; Afzal et al., 2014). The contribution of the phyllosphere microbiome to air pollutant degradation via oxidation mechanisms has been highlighted recently in Weyens et al. (2015). Furthermore, outdoor plants are a useful source of microbes for local dispersal and emigration to the built environment (Miletto and Lindow, 2015; Lymperopoulou et al., 2016; Leung et al., 2018). Sustaining a diverse microbiome on plants outdoors can have a potentially positive outcome on the health of citizens, similarly, to the role of plants indoor (Berg et al., 2014).

Despite the fact that the relationship between urban plants and their associated microbiota has many potential health benefits, only a few studies have attempted to explore the vast diversity of these communities. Still fewer studies have addressed the changes in *Platanus* leaf bacterial communities in response to air pollution (Zhang et al., 2015; Gandolfi et al., 2017). In complex environments like the city, it is unclear how microbial community dynamics vary in response to a multitude of changing environmental factors, the addition of human-associated microbes, and the rising level of exposure to aerosolized pollution.

To address this knowledge gap, this study seeks to characterize the *Platanus × hispanica* (colloquially referred to as London Plane) phyllospheric microbiome across a gradient of urbanization and traffic density and associated differences in local air quality, in the region of Hasselt, Belgium. A chronosequence sampling was performed in 2014 and 2016 to allow for a 2-year comparison. In addition, multiple metadata were gathered and measured such as ultrafine particles and black carbon to characterize the exposure of each location to common representative components of vehicular-related air pollution.

## Materials and Methods

### Sample Collection

**FIGURE 1:**
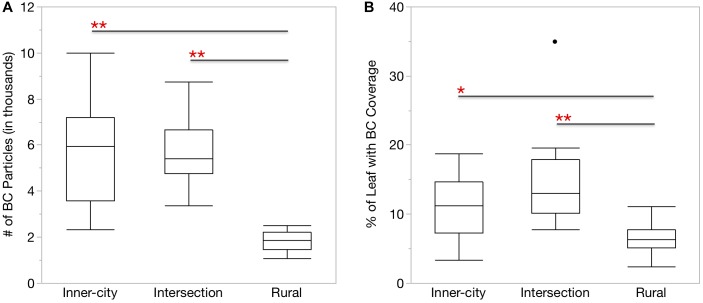
More BC particles were found on the surface of urban *Platanus × hispanica* leaves. Box plots show **(A)** the number of BC particles per mm^2^ leaf segment and **(B)** the percentage of the total leaf area covered by BC particles. ^∗^*p* < 0.01, ^∗∗^*p* < 0.0001. Box plots display the median, the first and third quartiles, and the maximum and minimum *n* = 18.

*Platanus × hispanica* trees at three locations were chosen around Hasselt, Belgium for sampling based on the local urban characteristics and adjacent traffic patterns, which were observed visually. Hasselt is a city of 102.24 km^2^ with a population of 77.000 inhabitants. Live air pollution data are monitored and available on the VMM website. The year average PM2.5 concentrations are 15 μg/m^3^ (VMM). Mothers and their newborns in the city of Hasselt and nearby city of Genk have the ability to participate in the ENVIR*ON*AGE birth cohort study, in which the effect of UFP exposure and black carbon on human health is investigated (Janssen et al., 2017, see also Figure 1 therein for a Corine land cover and main road map of Hasselt). At each of the three locations, adult trees were sampled (see Google street view for the general tree morphology and environment). The “Rural” tree (50.969 N; 5.408 E) was located in the arboretum of Domein Bokrijk which has a low burden of urbanization and road traffic. The “Intersection” tree (50.930 N; 5.360 E) was located at a major intersection outside of the city proper; it represents lower urban intensity and has a high road traffic burden. The “Inner-city” tree (50.932 N; 5.347 E) was located within the city proper; it represents a higher urban intensity but has a lower road traffic burden compared to the Intersection. None of the trees chosen receive any type of annual maintenance such as pollarding or crowning.

In early May 2014 and 2016, we collected leaves with ethanol sterilized gloves from four equidistant points around each tree at an approximate height of 3–4 ms, which was judged by the length of our cutting tool and near to the bottom of all trees sampled. Each of the four replicate samples consisted of three leaves from the same stem and weighed approximately 2.5 g. The sample leaves were transferred into sterile falcon tubes with 20 ml of 10 mM MgSO_4_ solution and transported to lab.

At each location, ultra-fine particles (UFP, < 1.0 μm diameter) were measured with an Aerasense Nanomonitor (Philips, Amsterdam, Netherlands) during the same hours as the leaf sampling took place and additional leaves were collected to determine their black carbon (BC) content. We took the land-use classifications for each location from the 2012 CORINE Land Cover database (European Union, 2018).

### Black Carbon Content Determination

#### Image Acquisition

The black carbon (BC) content of the leaves was determined by a specific and sensitive technique especially designed to quantify carbonaceous particles based on the non-incandescence-related white-light generation of those particles under femtosecond pulsed illumination as published before (Bové et al., 2016). For this, a Zeiss LSM 510 META NLO (Carl Zeiss, Jena, Germany) mounted on an Axiovert 200M equipped with a two-photon femtosecond pulsed laser (MaiTai DeepSee, Spectra-Physics, United States; 810 nm, 150 femtoseconds, 80 MHz, 10 mW laser power on the sample) was used. Three, 1 × 1 cm^2^ squares were cut from the left, right, and top of two leaves for each location and taped on coverslips with the adaxial side facing downward for imaging. Z-stacks were taken, using a 10x/0.3 (Plan-Neofluar) objective, throughout the plant tissue sections containing approximately 25 images with a size of 900 × 900 × 318 μm^3^ (1.8 × 1.8 × 6.6 μm^3^ pixel size and 3.2 μs pixel dwell time). White-light from the BC particles was acquired in the non-descanned mode after spectral separation and emission filtering employing a 400–410 nm band-pass filter. Simultaneously, the autofluorescence from the leaves were captured using a 450–650 nm band pass filter. Three randomly chosen z-stacks are made per tissue biopsy and three biopsies are taken per leaf and two leaves per location. Hence, in total per location, 18 technical replicates were included in the analysis.

#### Image Analysis

The number and total area of BC particles in the acquired z-stacks were determined using an automated and customized Matlab program (Matlab, 2013, Mathworks, Netherlands). First, a maximum project of the *z*-stack is performed. Next a peak-find algorithm counts connected pixels above a threshold value of 1.28% (manually validated using Fiji; Image J v 2.0, Open source software), which removes background signals. In the end, two output metrics were defined: (i) the total number of detected BC particles per square millimeter leaf and (ii) the percentage of leaf area covered with particles, i.e., total area of BC particles normalized over the imaged area of the leaf.

### Sample Preparation and DNA-Extraction

To release the epiphytic bacteria from the surface, sample leaves in 20 ml of 10 mM MgSO4 were vortexed on high for 2 min. The leaves were removed and the epiphyte solution was split into three aliquots which were passed through three sterile 0.2 μm nitrocellulose filters (Merck, Darmstadt, Germany). Each filter was transferred to a separate 2 ml bead beating tube with 0,1 mm glass beads (MoBio, Carlsbad, CA, United States) to release the epiphytes by shaking into Lysis Buffer P solution from the Invisorb Spin Plant Mini Kit (Stratec Molecular, Berlin, Germany). The lysis buffer was transferred to a new tube and gDNA was extracted with the kit according to manufacturer’s instructions, utilizing the Prefilter to remove excess glass beads and plant material.

### Preparation of 16S Library for Sequencing With Illumina MiSeq

#### Amplification of Target Sequences

The three gDNA extraction replicates obtained from each leaf sample were amplified separately with the following 16S rRNA gene primers. The first round of PCR targeted the V5–V7 region with primers 799F (5′-AACMGGATTAGATACCCKG-3′, Chelius and Triplett, 2001) and 1391R (5′-GACGGGCGGTGWGTRCA-3′, Walker and Pace, 2007) to minimize amplification of non-target rDNA from the leaf cells (Beckers et al., 2016). Initial denaturation at 95°C for 2 min, 32 cycles of 95°C for 30 s, 55°C for 90 s, 72°C for 45 s, and a final extension of 72°C for 7 min. Round one PCR products were separated on a 1.5% agarose gel to exclude the larger mitochondrial 18S fragment (∼1000 bp) and leftover primers. The 16S bands were removed with x-tracta^TM^ Gel Extractors (Promega Corporation, Madison, WI, United States) and cleaned with QIAquick Gel Extraction Kit (Qiagen, Venlo, Netherlands).

A second, shorter PCR used 2–4 μl of the previous amplicons to target the same region with 799F-ad (5′-TCGTCGGCAGCGTCAGATGTGTATAAGAGACAGAACMGGATTAGATACCCKG-3′) and 1193R-ad (5′-GTCTCGTGGGCTCGGAGATGTGTATAAGAGACAGACGTCATCCCCACCTTCC-3′, based on Bodenhausen et al., 2013). Primers were obtained from Biolegio (Nijmegen, Netherlands) with the Illumina adaptors added to the 5′ end, primer sequence region is underlined. Initial denaturation at 95°C for 2 min, 25 cycles of 95°C for 30 s, 55°C for 30 s, 72°C for 45 s, and a final extension of 72°C for 5 min. Fast Start HiFi (Roche Applied Science, Mannheim, Germany) components were used for all PCR amplifications. 0.5 and 0.4 μM of each primer was used in the first and second PCR, respectively. 25 μl reaction volumes had 1× reaction buffer with 1.8 mM MgCl2 included, 0.2 mM of each Roche dNTP, and 1.5 U Roche High-Fidelity Taq polymerase.

#### Preparation of the 16S Amplicon Library

The amplicons resulting from each set of extraction replicates (*n* = 3) were combined prior to barcoding the samples. Unique combinations of Nextera XT index kit (Illumina, San Diego, CA, United States) were attached to the 5′ and 3′ ends of each individual library, following the method used by Coretti et al. (2017). Subsequently, the barcoded samples were cleaned-up with AMPure XP beads (Agencourt Bioscience, United States) and the individual DNA libraries were quantified with Quantifluor dsDNA (Promega Corporation, Madison, WI, United States). Equimolar concentrations of each sample were pooled, and the final pooled library was sent to Macrogen (Macrogen Korea, Seoul, South Korea) for sequencing on the Illumina MiSeq platform with a 300 bp paired-end protocol.

### Data Analysis of 16S Amplicon Sequences

Prior to delivery, sequences were trimmed by Macrogen with Scythe (v.994) and Sickle to remove the adapter sequence and reads less than 36 bp. Remaining reads were then quality filtered and chimera checked with DADA2 v 1.8 (Callahan et al., 2016). The resulting ASV table and representative sequences were imported as artifacts to Qiime 2 (Gregory Caporaso et al., 2010). To improve the accuracy of taxonomic classification, we extracted our 16S target region of 799F–1193R from the Silva v132 (Quast et al., 2013) 99% data set and used these optimized reference sequences to train a custom Naïve Bayesian classifier on the majority_taxonomy_7 SILVA database. FastTree 2 (Price et al., 2010) generated rooted and unrooted phylogenetic trees after masked alignment of the reference sequences. All samples were rarefied to the lowest equal number of reads and we checked for adequate sampling depth with the Good’s Coverage metric. Genera occurring in at least 75% of the replicate samples each year were included as part of each tree’s persistent microbiome.

### Statistical Analysis

#### Bacterial Taxonomic Diversity

Intra-tree diversity was assessed with ASV observations, Shannon diversity, Simpson diversity, and Faith’s phylogenetic diversity. Differences in intra-tree diversity between locations was tested with Kruskall–Wallis Rank Sum Test followed by the Pairwise Wilcoxon Rank Sum Test. Singletons were removed from the ASV table for all following analysis. Inter-tree diversity was assessed in R (R Development Core Team, 2018) utilizing the ape, phyloseq, ggplot2, vegan, RVAideMemoire packages (Paradis et al., 2004; McMurdie and Holmes, 2014; Oksanen, 2015; Wickham, 2016; Hervé, 2018). Bacterial community diversity was measured with the Bray-Curtis dissimilarity metric and visualized on a PCoA plot. We compared the community differences between tree locations and years with the adonis function from vegan (999 permutations), followed by the pairwise.perm.anova function from RVAideMemoire with FDR correction (999 permutations).

#### Bacterial Functional Diversity

The web-based metagenome inference tool Piphillin (Iwai et al., 2016) was used at the 97% identity cut-off to normalize 16S ASV counts and assign KEGG orthologs and pathways (KEGG, May 2017 Release). Bacterial taxonomy and inferred KEGG pathways were analyzed with the Linear discriminant analysis Effect Size (LEfSe) tool (Segata et al., 2011) hosted on the Huttenhower Galaxy server (Afgan et al., 2018) to discover significant associations between the land class types. Pairwise comparisons were made between years and the Kruskal–Wallis sum rank and Wilcoxon signed rank-sum tests were both run at *p* < 0.05. We also tested for differential abundance of the inferred KEGG orthologs between all groups using DESeq2 (Love et al., 2014) hosted on the Freiburg Galaxy server (Afgan et al., 2018). Orthologs were rounded to the nearest integer. Significant differences are those less than 0.05 after a Benjamini-Hochberg adjustment, which controls for false discoveries.

#### Air Quality Characteristics

We compared the average ultra-fine particle concentration and size with ANOVA and *post hoc* Tukey-Kramer HSD tests. Black carbon particles and leaf coverage were compared with the Kruskal-Wallis Rank Sum Test followed by the Pairwise Wilcoxon Rank Sum Test. All tests were done in JMP Pro 13 software (SAS Institute Inc., Buckinghamshire, United Kingdom).

### Data Deposit in NCBI

All raw Illumina 16S rRNA gene sequencing data were submitted to NCBI SRA with project identifier PRJNA505409, and sample IDs SRX5015223 to SRX5015246.

## Results

### Sampling Site and Air Quality Characteristics

To better understand the bacterial phyllosphere of *Platanus × hispanica* trees planted in urban centers and the effects which anthropogenic pressures may exert on these communities, we chose three locations of different urban density and traffic patterns from which to sample air quality and leaves. Hasselt is the capital city of the Limburg province in Belgium. According to open data on statbel.be, between 2014 and 2016, the municipal population rose from 76,000 to 77,000 inhabitants. Table 1 further describes the sampling sites, including the name we assigned each location to help differentiate between the trees, the coordinates and land use class in which each tree stands, and the distance from each tree to the nearest road. The average UFP per cm^3^ and average UFP size in nanometers are also listed in Table 1.

**Table 1:** Characteristics and UFP measurements at each of the *Platanus × hispanica* sampling sites.

Location ID	Coordinates	Land-use Designation^a^	Distance to nearest road (m)	Average UFP per cm^3^	Average UFP size (nm)
Rural	50.969 N - 5.408 E	Mixed Forest	400	3,689	79
Inner-city	50.932 N - 5.347 E	Discontinuous Urban Fabric	5	14,451	72
Intersection	50.930 N - 5.360 E	Discontinuous Urban Fabric	5	14,208	65

*^a^ Land-use Designation comes from the 2012 CORINE Land Cover database.*

Air sampled at the urban locations, i.e., the inner-city and intersection sites, had significantly higher concentrations of UFP on average than the rural location (*p* < 0.001), but with no significant difference between each other. However, particle size did change based on location. Compared to the inner-city site, the rural position had significantly larger particles (*p* < 0.001) and the intersection location had significantly smaller particles (*p* < 0.001).

Similar to the UFP in the air, the urban leaves also had more BC particles than the rural leaves (Figure 1A). When accounting for the difference in surface area, the urban leaves also had a greater percentage of area covered by the BC particles (Figure 1B).

### Phyllospheric Bacterial Community Structure

After quality filtering, our 16S amplicon data set consisted of 178,908 sequences, ranging from 2,907 to 34,895 sequences per sample. In an effort to keep all 24 samples in the dataset, rarefaction was set to the lowest number of reads and subsequently checked with Good’s coverage index. The average sample coverage was 0.99 (± 0.001 SE), which was deemed sufficient to continue with all samples included in further analysis.

The rarefied dataset represented bacterial communities from 17 phyla, 273 identified genera, and 1,502 ASVs. Overall, the most dominant phyla were Proteobacteria (71.5%), Deinococcus-Thermus (16.9%), Actinobacteria (4.5%), and Firmicutes (1.9%). These phyla were present on all trees in both sampling years and except for the Firmicutes which were missing in three of the eight Rural samples, these phyla were present in all samples.

**FIGURE 2:**
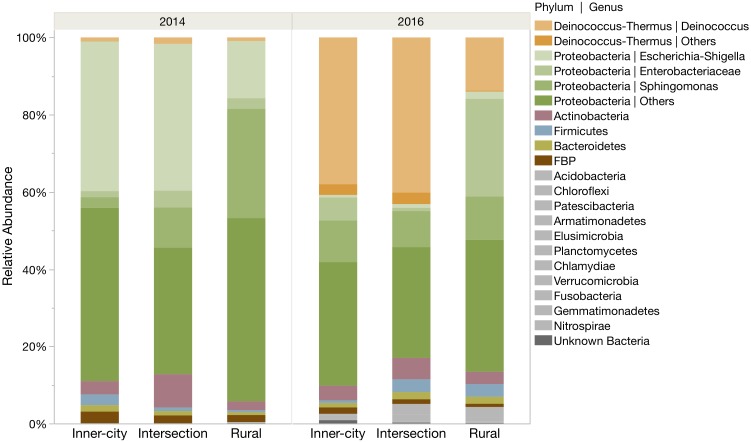
Relative abundance of ASVs for each location in both sampling years. ASVs are grouped into phyla and several prominent genera are highlighted within their phylum. The six most abundant phyla are color coded and the remaining twelve rare phyla are grouped as gray bars at the bottom.

Proteobacteria was the most abundantly identified phylum at all locations, however the phylum was more abundant in 2014 compared to 2016 (Figure 2). In 2014, Proteobacteria represented 93.4 and 86.8% of the rural and urban bacterial community, respectively. By 2016, the community structure had shifted to include more Deinococcus-Thermus bacteria, especially on the urban tree leaves (42.0%) compare to the rural tree leaves (14.0%). Even so, the Proteobacteria phylum still made up the majority of bacteria at each location. Other highly abundant phyla include the Actinobacteria (2.3–6.0%), Firmicutes (0.6–3.3%), Bacteroidetes (0.7–1.8%) and FBP (0.8–2.5%).

A closer look into the community structure at the level of genus illustrated not only the same temporal differences we saw on the phyla level, but also interesting similarities between the urban samples that are not in the rural samples (Figure 2). In 2014, the most abundant bacterial genus on the urban leaves was *Escherichia*-*Shigella* and in 2016 it was *Deinococcus* with an average relative abundance of 38.8% (±0.5 SE). The most abundant genus of the rural tree in 2014 was *Sphingomonas* (28.3%) and in 2016, unknown Enterobacteriaceae (25.3%). The top ten most abundant genera at each tree account for more than three-quarters (78.7% ± 2.5 SE) of the total genera.

### Persistent Bacterial Genera of the Core *Platanus × hispanica* Leaf Microbiome

**FIGURE 3:**
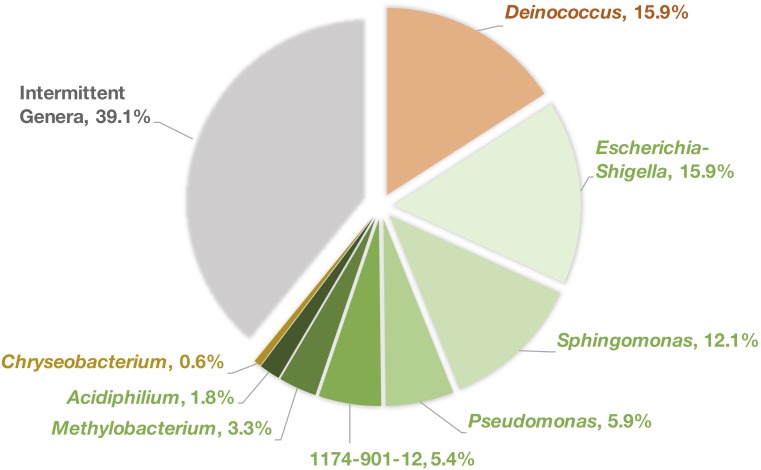
Percentage of reads attributed to each of the shared persistent genera. Colors refer to the corresponding phylum for each genus, orange = Deinococcus-Thermus, green = Proteobacteria, gold = Bacteroidetes, gray = all other intermittent bacterial genera.

At each tree location, bacterial genera that were persistently identified in the phyllosphere from one sampling year to another were compared between all three locations and we found eight genera are consistently shared across all trees (Figure 3). While this represents a small fraction of the overall diversity (28% of all ASVs) it comprises a large portion (60.9%) of the total reads.

The shared genera were *Deinococcus*^∗^ (15.9% of total reads), *Escherichia-Shigella* (15.9%), *Sphingomonas*^∗^ (12.1%), *Pseudomonas* (5.9%), 1174-901-12^∗^ (Family: Beijerinckiaceae, 5.4%), *Methylobacterium*^∗^ (3.3%), *Acidiphilium* (1.8%), and *Chryseobacterium* (0.6%). Of these six, only four genera, those denoted by “^∗^,” were identified in 100% of the samples taken.

Between the intersection and inner-city sites, *Friedmanniella* was a persistently identified genus and between the intersection and rural leaves, *Afipia* was also persistent. The rural and inner-city locations did not share any more genera than those common to all three sites. However, the rural and inner-city leaves each had one genus unique to that location while the intersection leaves had four.

### Diversity of the Bacterial Communities

There was no meaningful difference in observed species or phylogenetic diversity between the locations (Table 2). Although in 2014 the inner-city had slightly less diversity than the rural leaves (Simpson and Shannon *p* = 0.0304), this was not the case in 2016. The phylogenetic diversity (Faith’s PD) and observed ASV counts did not differ by location in both years.

**Table 2:** Species and phylogenetic diversity measures of the *Platanus × hispanica* bacterial phyllosphere communities.

	2014			2016		
	Rural	Inner-city	Intersection	Rural	Inner-city	Intersection
Obs. ASVs	79.0 ± 6.2	65.5 ± 11.3	72.5 ± 6.1	150.3 ± 38.8	111.5 ± 15.6	233.8 ± 73.2
Simpson	0.966 ± 0.001	0.927 ± 0.007	0.945 ± 0.014	0.962 ± 0.022	0.962 ± 0.009	0.978 ± 0.009
Shannon	5.59 ± 0.06	4.58 ± 0.16	5.25 ± 0.25	6.00 ± 0.60	5.79 ± 0.13	6.78 ± 0.42
Faith’s PD	7.59 ± 0.79	7.93 ± 0.80	8.13 ± 0.34	13.30 ± 1.98	11.44 ± 1.26	19.36 ± 3.21

*Mean ± Standard Error, n = 4.*

**FIGURE 4:**
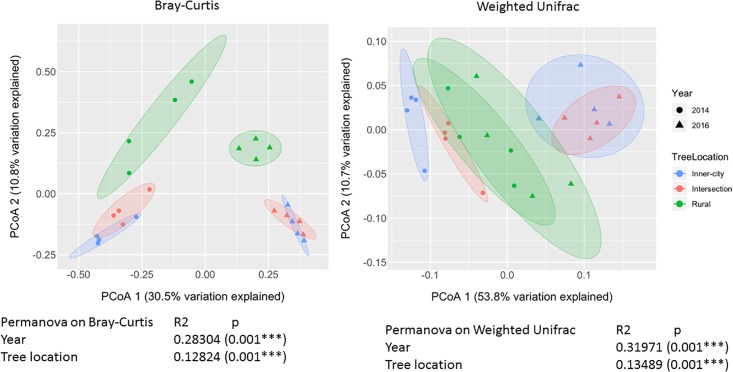
PCoA plot of Bray-Curtis dissimilarity values and Weighted Unifrac between epiphytic bacterial communities on *Platanus × hispanica* leaves. Circles = 2014, triangles = 2016. Blue = Inner-city, red = Intersection, and green = Rural. Each normal data ellipse is drawn at a level of 0.80.

To compare the different bacterial composition of each leaf phyllosphere by location and between years, a Bray-Curtis dissimilarity matrix was calculated for each sample. PCoA plotting (Figure 4) revealed that the communities are differentiated by year (*p* = 0.001), as seen on the first axis with 30.5% of the variation. Since most of the variation is explained by the first axis, we employed a blocking strategy to separate the years before testing for significance of community dissimilarity between tree locations (*p* = 0.001). Pairwise PERMANOVA analysis (Table 3) of the 2014 samples revealed the urban bacterial communities of inner-city and intersection were dissimilar (*p* = 0.032). But in 2016, the two urban communities clustered together (*p* = 0.461). But what the urban leaves both had in common was that they were significantly different than the rural leaves in both sampling years (2014, *p* = 0.032 and 2016, *p* = 0.045).

**Table 3:** *p*-value of each pairwise PERMANOVA (with FDR correction).

	2014	2016
Intersection – Inner-city	0.032	0.461
Intersection – Rural	0.032	0.045
Rural – Inner-city	0.032	0.045

### Linear Discriminant Analysis Effect Size Reveals Differential Abundance of Bacteria Between Land Class Types

**FIGURE 5:**
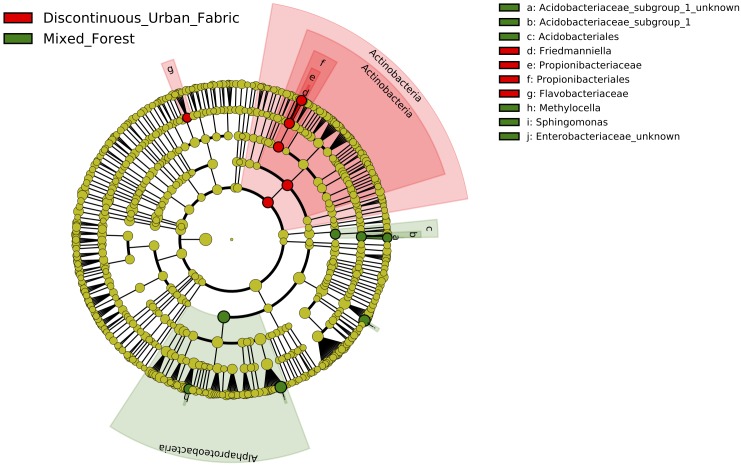
Cladogram representing the LEfSe results of which taxa are enriched in the urban (red) and rural (green) bacterial communities. LDA inclusion threshold set at 2.0.

Controlling for difference between the years again, we were able to determine that land use class type had an effect on the relative abundance of two major bacterial classes in the phyllosphere. LDA inclusion threshold > 2.0, *p* < 0.05. The LEfSe cladogram of Figure 5 shows Actinobacteria and more specifically, Propionibacteriales, Propionibacteriaceae, and Friedmanniella, in addition to Flavobacteriaceae are more enriched on the urban leaves. Whereas Alphaproteobacteria, *Methylocella*, *Sphingomonas*, Acidobacteriales, Acidobacteriaceae Subgroup 1, and unknown Enterobacteriaceae are more enriched on leaves in the rural environment.

### Predicted Functional Profile for Epiphytic Bacteria

**FIGURE 6:**
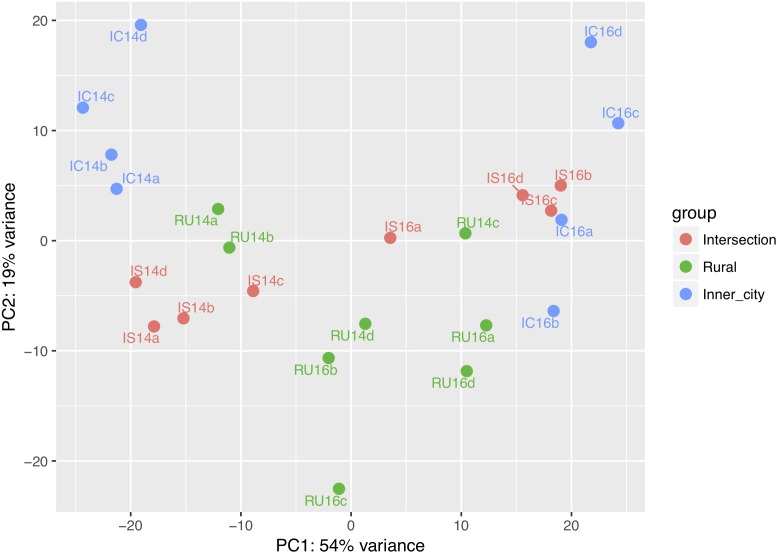
PCoA plot for the similarity of predicted KEGG orthologs in each sample. IC = Inner-city, IS = Intersection, RU = Rural, 14 = 2014 and 16 = 2016. The lowercase letters refer to one of the four replicates from each group.

Across all samples, 6,846 orthologs and 304 pathways were identified using the KEGG reference database. The similarity between predicted KEGG orthologs was visualized on a PCoA plot (Figure 6). Comparable to the Bray-Curtis dissimilarity of bacterial communities (Figure 4), the first axis differentiates between the years and the second axis distinguishes variation between the locations.

In order to determine how the functional profile of the microbiome may have changed over time, we compared the percentage of orthologs with significantly different abundances between 2014 and 2016 (Table 4). The rural tree remained relatively stable with 10.75% of the total orthologs changing between the years. In contrast, the inner-city and intersection trees were more dynamic; roughly half of the orthologs had significantly different abundances (Inner-city, 52.05%; Intersection, 50.18%).

**Table 4:** Percentage of KEGG orthologs with significantly different abundances (*p* < 0.05) between years at each sampling location.

	Inner-city	Intersection	Rural
2014–2016	52.02%	50.18%	10.75%

To determine if the functional profile of the microbiome was influenced by location, we looked at the inferred orthologs within each year (Table 5). The percentage of orthologs with significantly different abundances was consistent between years for the inner-city and rural trees (2014, 21.22%; 2016, 20.92%). But their relationship to the intersection tree had more variation.

**Table 5:** Percentage of KEGG orthologs with significantly different abundances (*p* < 0.05) between locations in each year of sampling.

	2014	2016
Intersection – Inner-city	12.84%	0.02%
Intersection – Rural	10.99%	17.00%
Rural – Inner-city	21.22%	20.92%

**FIGURE 7:**
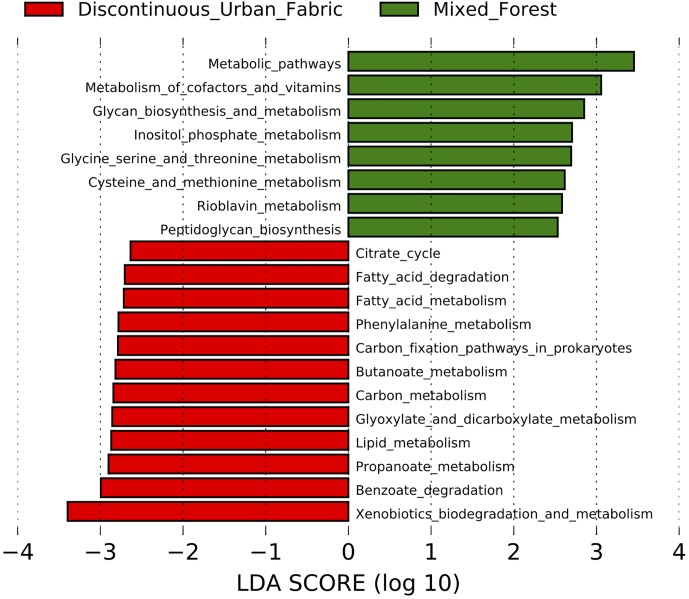
LDA scores of the metabolic pathways enriched in the urban (red) and rural (green) epiphytic phyllosphere bacterial communities. Inclusion threshold set at an LDA score of 2.5.

To determine if the urban phyllosphere might be metabolically enriched in alternative carbon sources, such as polycyclic aromatic hydrocarbons, we used the LEfSe tool to analyze the 154 KEGG annotated metabolic pathways predicted by Piphillin. The enriched pathways (*p* < 0.05) and corresponding LDA effect size scores for each land use class type are displayed in Figure 7. The bacteria on leaves in the mixed forest environment were more associated with pathways concerning “metabolism of cofactors and vitamins” and “glycan biosynthesis and metabolism,” while the urban communities are predicted to contain more xenobiotics and lipid metabolism pathways.

## Discussion

In this study we used culture-independent methods to compare the epiphytic bacterial communities on *Platanus × hispanica* leaves between rural and urban locations to address what effects the local air quality might have on diversity, taxonomic composition, and potential functionality. As various studies of trees in tropical and temperate climates have shown, epiphytic bacterial communities are often more similar on trees of the same species than on other trees in the same location (Redford et al., 2010; Kim et al., 2012; Kembel et al., 2014; Laforest-lapointe et al., 2016). Therefore, we chose to study only *Platanus × hispanica* trees in a gradient of urbanization and traffic density and where global meteorological trends were similar. In addition, samples were taken from the same trees in two non-consecutive years to consider if a core group of shared bacteria would be present.

### Air Quality Characteristics

From the perspective of microorganisms, the leaf habitat is vast in space but limited in nutrients and constantly exposed to environmental stresses like temperature fluctuations, UV radiation, and rainfall. In particular, leaf associated bacteria on trees in urban areas and close to roadways are recurrently exposed to aerosolized pollutants dispersed into the environment via vehicular exhaust (Weyens et al., 2015). As the majority of aerosolized particulate matter in urban environments is comprised of particles in the ultra-fine range of 100 – 10 nm, we chose to focus on this fraction as well (Morawska et al., 1998; Junker et al., 2000). Our results indicate the average concentrations of particles measured in this study (Table 1) are similar to those published in other European studies of urban and residential areas (Matson, 2005; Puustinen et al., 2007; Pey et al., 2009; Hofman et al., 2016).

In addition, we also measured BC directly on the leaf surface. BC is a common component of PM and UFP formed during incomplete combustion and aerosolized in automobile exhaust. Recently, the BC fraction of particulate matter has come under greater scrutiny for its negative effects on human health and the environment (Janssen et al., 2012; De Prins et al., 2014). Zhu et al. (2002) reported that UFP and BC concentrations were both highest next to roadways, therefore we expected to find more BC on the urban leaves as a result of their increase exposure to UFP and close proximity to roads (Table 1). This was confirmed by our results of the higher number of BC particles (Figure 1A), on urban leaves as well as the greater percentage of leaf coverage by the BC particles (Figure 1B). Although the Intersection has more traffic passing by than the Inner-city location, it did not make a significant difference in average UFP counts or BC accumulation on leaves. The abundance of smaller UFP at both of the urban locations and especially the more residential Inner-city location, is of concern as this size fraction is associated with negative effects on respiratory health (Leitte et al., 2011; Li et al., 2016).

### Abundant Bacteria in the *Platanus × hispanica* Phyllosphere

Proteobacteria, Actinobacteria, Firmicutes, and Bacteroidetes, that were the dominant phyla observed in our samples (Figure 2), are often reported in culture-dependent and culture-independent studies of leaf epiphytes. As expected, Proteobacteria was the dominant bacterial phylum in all samples, which is in accordance to numerous other studies on the bacterial community composition of plant leaves (Redford et al., 2010; Bodenhausen et al., 2013; Kembel et al., 2014; Laforest-lapointe et al., 2016; Lymperopoulou et al., 2016; Smets et al., 2016; Gandolfi et al., 2017). It has been suggested that this is due to the speed at which many bacteria within the Proteobacteria phylum replicate in comparison to other phyla (Leff et al., 2015).

Recently, bacteria within the phylum of Deinococcus-Thermus have also been described as typical phyllosphere residents (Redford et al., 2010; Laforest-lapointe et al., 2016). This might be due to a change in the methodology for studying aerial sections of the plant. Clone libraries and amplicon strategies not including the 799F primer for chloroplast exclusion (Chelius and Triplett, 2001) seem to be biased against this phylum (Redford and Fierer, 2009; Jackson and Denney, 2011; Beckers et al., 2016; Gandolfi et al., 2017). Indeed, the use of 799F–1115R primers found Deinococcus-Thermus on leaves of nearly all the 56 of the tree species studied (Redford et al., 2010).

Only two Deinococcus-Thermus genera, *Deinococcus* and *Truepera*, were identified in our samples. Of these, *Deinococcus* was more abundant on all leaves and in both years. This is not surprising as *Deinococcus* species are reportedly highly resistant to UV radiation and desiccation, which are fairly common environmental stressors to the phyllosphere. There are indications that *Deinococcus* might be useful for degradation of aromatic compounds. *Deinococcus* bacteria capable of phenanthrene degradation has been isolated from Ixora leaves in Thailand (Waight et al., 2007).

Similar to Deinococcus-Thermus, the candidate phylum FBP is also associated with extreme environments and often found in 16S rRNA gene sequencing of phyllosphere samples (Laforest-lapointe et al., 2016; Ruiz-Pérez et al., 2016). However, not much is known about these candidate bacteria since only one has been successfully cultured and studied (Tahon et al., 2018).

### Persistent Core Microbiome

Despite the dynamic and complex nature of host-associated microbiomes, it has been theorized that a core portion of each community is persistent across time and habitats (Shade and Handelsman, 2012). Members of the core microbiome are credited with being an integral part for the optimal microbial functioning needed to promote the health of their host-species (Toju et al., 2018).

In this study, we identified which genera were persistently found at each location and then compared these to find the shared persistent bacteria. We chose to compare genera rather than ASVs in order to include the possibility of functional redundancy within the same genus. As deciduous trees grow new leaves every year and the order of bacterial colonization matters (Maignien et al., 2014), it may not be reasonable to expect the same ASVs but to consider that ASVs of the same phylogenetic lineage will share the desired functional trait.

Although it is likely that the eight shared genera (Figure 3) are connected to the core microbiome because they were detected the leaf bacterial community in two non-consecutive years, due to sampling limitations, it is still unknown if they are universal to all *Platanus* trees or they are localized to this region and climate. Only one other study has sought to evaluate the taxonomic composition of bacterial communities on *Platanus* leaves (Gandolfi et al., 2017) for which we can compare our results. Out of the eight genera identified in our study, only Sphingomonas, Pseudomonas, and Methylobacterium were also consistently identified on *Platanus × acerifolia* leaves in Milan (Italy) over one growing season. Therefore, these three genera stand out as the most probable members for the core microbiome of *Platanus* leaves. It should be noted that *Platanus × acerifolia* and *Platanus × hispanica* are historically synonymous names for the same tree (Besnard et al., 2002) and bacterial communities on leaves of the two other common Platanus species, *orientalis* and *occidentalis*, have not yet been reported.

### Intra-Tree and Inter-Tree Diversity

The decrease in available green space in cities is often associated with negative effects on human health, mostly related to mental and physical wellbeing (Jim et al., 2018). In addition to the benefits received from eukaryotic diversity, exposure to a variety of prokaryotes is connected with establishing and maintaining a healthy immune system (Haahtela et al., 2013; Rook, 2013). We clearly see that the built environment limits plant and animal biodiversity in cities, so there is concern regarding the loss of microbial richness, which is more difficult to assess.

Phyllospheric communities frequently undergo disturbances such as changes in UV radiation, precipitation, and resource availability that disrupt the natural community succession (Redford and Fierer, 2009). The significant temporal difference in intra-tree diversity between 2014 and 2016 is likely to be a combination of multiple environmental factors. Therefore, within each year we sampled from the same trees located in close proximity to each other so meteorological changes would have less of an effect on location. Based on a comparison with the leaves of the trees in the urban and rural areas, our results indicated that the richness and diversity of bacteria on urban *Platanus × hispanica* tree leaves are not significantly impacted by increased exposure to UFP and BC (Table 2). This fits with other similar studies on urban phyllospheric bacterial diversity (Smets et al., 2016; Laforest-Lapointe et al., 2017).

Redford and Fierer (2009), Laforest-lapointe et al. (2016), and Gandolfi et al. (2017) all found that over one growing season, the influence of site was a stronger driver of inter-tree compositional diversity for the phyllosphere than temporal changes. We chose to study a longer separation of time, where the trees have lost and regained their leaves twice, to address the impact of site on the establishment of new bacterial communities rather than its effect on the community succession.

Our results indicate that the taxonomic composition of epiphytic bacterial communities is influenced by urbanization. It was apparent that each community saw a large temporal shift in bacterial taxa, but within each year the general relationships between communities remained nearly the same (Figure 4).

### Differential Abundant Genera Between Locations

Consistent in both sampling years, the urban trees shared a similar percentage of the same dominant genus (Figure 2). In 2014, this was *Escherichia-Shigella* and in 2016, it changed to be *Deinococcus*. Interestingly, when considering changes in relative abundance at the urban trees, *Deinococcus* was infrequently identified in 2014 and *Escherichia-Shigella* was, in turn, similarly, scarce in 2016. The rural tree had a distinctly different pattern of abundant bacteria compared to the urban trees in both sampling years.

The bacteria revealed by LEfSe analysis to be enriched at the urban and rural locations in both years are mostly those that are typically found in environmental samples (Figure 5). The abundance of Propionibacteriaceae on the urban leaves stands out however, as this family is often associated with human skin. Lehtimäki et al. (2017) found *Friedmaniella* was enriched on the forearms of children living in urban areas compared to those in rural homes. Also of note is that although the *Sphingomonas* genus was persistently identified on all *Platanus × hispanica* leaves (Figure 3), it was shown here to be more commonly identified in the rural samples.

### Functional Profiles

We wanted to know if the temporal and spatial differences in taxonomic composition (Figure 4) would also be reflected in the inferred functional composition (Figure 6). For this purpose, we employed Piphillin because it has the flexibility to interpret inferences from ASV tables rather than needing a closed reference OTU picking strategy. One of the unfortunate disadvantages is that Piphillin is less accurate than PICRUSt and Tax4fun when used on environmental samples (Iwai et al., 2016). Hopefully this will change in the future when more genomes of environmental microbes are fully sequenced and added to databases. It is also important to note, that although the functional orthologs and pathways are directly inferred from the taxonomic composition of each bacterial community, Piphillin uses a reference database to correct for variation in 16S copy number and therefore the functional differential abundance cannot be directly compared to the taxonomic differential abundance.

The KEGG orthologs predicted for 2014 were compared to those predicted for 2016 at each location in order to bring a greater understanding of how the functional aspect of each bacterial community changed over time (Table 4). The urban trees saw a larger change in ortholog composition between the sampling years than the rural tree. This suggests that even though the taxonomic composition of the rural tree was significantly different, the predicted functional profile of the bacteria remained similar.

The difference in ortholog composition between locations (Table 5) also mirrors the differences seen in taxonomic compositions. In both sampling years, roughly 21% of the rural and inner-city orthologs had significantly different abundances. However, the dissimilarity between each of those bacterial communities to the intersection community was more dynamic across both years. In terms of geographic distance, rural and inner-city are also further away from each other and experiencing other environmental conditions (neighboring plants versus more artificial city surfaces), so a higher percentage of differently expressed KEGG orthologs suggest that the communities exert different metabolic functions. In order to confirm this, a shotgun sequencing approach, metabolomics study or individual-based microbial ecology (IBME) should be performed to quantify the performance of microorganisms in their natural habitats. An IBME approach has confirmed that heterogeneity in fructose availability has an important role in the reproductive success of bacterial immigrants to the leaf surface (Remus-Emsermann and Leveau, 2010). How atmospheric car-exhaust related air pollutants might affect carbon substrates in the phyllosphere and therefore sustain metabolically different microbial communities remains to be explored.

We limited the LEfSe analysis (Figure 7) to include only the metabolic pathways as our interest was mainly in determining if the urban trees had a greater inclination toward accumulating bacteria with pathways to degrade common traffic related compounds, such as polycyclic aromatic hydrocarbons. While many of these were not directly enriched, LEfSe did reveal that overall degradation of xenobiotic compounds was predicted to be more common in the urban phyllosphere bacterial communities.

The benzoate degradation pathway is also enriched in the urban samples. This pathway is intimately involved with the degradation of monoaromatic hydrocarbons such as benzene and toluene, as well as degradation of polycyclic aromatic hydrocarbons metabolites. Although these results do not necessarily mean that the enzymes in this pathway are more active in the urban bacterial communities. It has been observed previously that bacteria with hydrocarbon degradation potential are inhabiting the phyllosphere of city plants, e.g., we isolated previously hydrocarbonoclastic *Rhodococcus* (Stevens et al., 2017b) and *Bacillus* (Stevens et al., 2017a) on *Hedera helix* leaves.

## Conclusion

The results of our biennial study on urban and rural leaf-associated epiphytic bacterial communities provide additional insights in the phyllosphere microbiome of *Platanus × hispanica* trees in the city. Bacterial communities on urban leaves are exposed to a greater concentration of harmful aerosolized pollutants from traffic exhaust compared to rural leaves. Urbanization alone was not a major factor explaining bacterial diversity in the phyllosphere and this is in line with previous studies on *Platanus* suggesting that seasonality acted as a stronger driver of alkane hydroxylase genes involved in the degradation of atmospheric hydrocarbons, rather than sampling location. Although we found large differences between the two sampling years, we consistently observed that human associated bacteria and degradation pathways for xenobiotic compounds are more likely to be enriched in the urban samples.

Because we performed a biennial study of the *Platanus* microbiome, we could determine that the patterns of dissimilarity in taxonomic composition between the sites were somewhat predictable. It also appears the taxonomic composition is dependent on more environmental factors than just the surrounding land use class designation.

Our study quantified part of the multi-complex effects of urbanization and associated air pollution on the *Platanus* phyllospheric microbiomes in Hasselt (Belgium) and how this may subsequently impact the health of urban inhabitants. Further research into the influence of urban microbiomes is certainly needed. Information on the temporal, geographical and functional variations, will improve our understanding of plant-microbe interactions and how we can manipulate these communities in future to scavenge air-borne pollutants in the city.

## Data Availability

The datasets generated for this study can be found in SRA NCBI, PRJNA505409.

## Author Contributions

JE and NW conceived the study design. JV, NW, and ST coordinated the execution of the project by JE. JE wrote this manuscript in collaboration with ST. HB performed the black carbon measurements on the leaves. JV promoted the study. All authors contributed to the elaboration of the study design and took part in reviewing the methods read and approved the final version of the manuscript.

## Conflict of Interest Statement

The authors declare that the research was conducted in the absence of any commercial or financial relationships that could be construed as a potential conflict of interest.
